# Zn^2+^-dependent surface behavior of diacylglycerol pyrophosphate and its mixtures with phosphatidic acid at different pHs

**DOI:** 10.3389/fpls.2014.00371

**Published:** 2014-07-29

**Authors:** Ana L. Villasuso, Natalia Wilke, Bruno Maggio, Estela Machado

**Affiliations:** ^1^Departamento de Biología Molecular, FCEFQN, Universidad Nacional de Río CuartoRío Cuarto, Argentina; ^2^Facultad de Ciencias Químicas, Departamento de Química Biológica-Centro de Investigaciones en Química Biológica de Córdoba, Universidad Nacional de Córdoba, Ciudad UniversitariaCórdoba, Argentina

**Keywords:** diacylglycerol pyrophosphate, phosphatidic acid, plant lipid signaling, membrane packing, glycerophospholipid monolayers, Zn^2+^

## Abstract

Diacylglycerol pyrophosphate (DGPP) is a minor lipid that attenuates the phosphatidic acid (PA) signal, and also DGPP itself would be a signaling lipid. Diacylglycerol pyrophosphate is an anionic phospholipid with a pyrophosphate group attached to diacylglycerol that was shown to respond to changes of pH, thus affecting the surface organization of DGPP and their interaction with PA. In this work, we have investigated how the presence of Zn^2+^ modulates the surface organization of DGPP and its interaction with PA at acidic and basic pHs. Both lipids formed expanded monolayers at pHs 5 and 8. At pH 5, monolayers formed by DGPP became stiffer when Zn^2+^was added to the subphase, while the surface potential decreased. At this pH, Zn^2+^ induced a phase transition from an expanded to a condensed-phase state in monolayers formed by PA. Conversely, at pH 8 the effects induced by the presence of Zn^2+^ on the surface behaviors of the pure lipids were smaller. Thus, the interaction of the bivalent cation with both lipids was modulated by pH and by the ionization state of the polar head groups. Mixed monolayers of PA and DGPP showed a non-ideal behavior and were not affected by the presence of Zn^2+^ at pH 8. This could be explained considering that when mixed, the lipids formed a closely packed monolayer that could not be further modified by the cation. Our results indicate that DGPP and PA exhibit expanded- and condensed-phase states depending on pH, on the proportion of each lipid in the film and on the presence of Zn^2+^. This may have implications for a possible role of DGPP as a signaling lipid molecule.

## INTRODUCTION

Phospholipids are mostly conceived as playing a structural role in lipid bilayers but important aspects on the implications of several phospholipids in lipid-mediated signal transduction have emerged over the past decade (Wang et al., 2006).

Diacylglycerol pyrophosphate (DGPP) is a minor phospholipid found in biological membranes, with a relatively simple chemical structure within the glycerophospholipid family (Wissing and Behrbohm, 1993a). Diacylglycerol pyrophosphate is synthesized from phosphatidic acid (PA) and ATP via the reaction catalyzed by phosphatidate kinase (PAK) and dephosphorylated to PA by the enzyme DGPP phosphatase (Wissing and Behrbohm, 1993b). The average concentration of DGPP in cell membranes is usually very low but evidence suggests that DGPP may act as a novel second messenger with important roles in diverse cellular processes in plants that are related to drought and osmotic stress or salinity (van Schooten et al., 2006). Diacylglycerol pyrophosphate formation is transient and it is always associated with variations of the amount of PA, therefore its synthesis may also be involved in attenuating PA levels (Munnik et al., 1996; van Schooten et al., 2006; Racagni et al., 2008; Paradis et al., 2011). The concentration of PA is maintained at low levels in the cell as a result of its continuous conversion into other lipid species (Arisz et al., 2009; Villasuso et al., 2013).

Phosphatidic acid, the lipid precursor of DGPP, is the glycerophospholipid with the simplest chemical structure in biological membranes; its behavior is crucial for cell survival since it is a phospholipid involved in the synthesis of phospholipids and triacylglycerols thus playing a focal role in cell signaling (Athenstaedt and Daum, 1999). Phosphatidic acid signaling acts by binding effector proteins and recruiting them to a membrane, which regulates the proteins’ activity in cellular pathways (Testerink and Munnik, 2011). Binding is mainly dependent on the concentration of the lipid in the bilayer and it depends on non-specific electrostatic interactions between clusters of positively charged amino acids in the protein and the negatively charged phosphomonoester head group of PA (Shin and Loewen, 2011).

Diacylglycerol pyrophosphate is an anionic phospholipid with a pyrophosphate group attached to diacylglycerol. It was shown that, depending on the pH, the pyrophosphate moiety of DGPP could display 2 or 3 negative charges making it a highly polar molecule (Villasuso et al., 2010; Strawn et al., 2012). Consequently, the ionization of the pyrophosphate group may be important for allowing electrostatic interactions between DGPP and proteins as well as with bivalent cations such as Zn^2+^ and Ca^2+^ (Han et al., 2001; Zalejski et al., 2006; Strawn et al., 2012). This can participate in regulating Zn^2+^-mediated enzyme activities (Han et al., 2001; Kim et al., 2013). Therefore, it is possible that PA and DGPP could be involved in Zn^2+^ binding thus affecting the lipid signal although the interaction between zinc and DGPP has not been directly demonstrated.

Zinc (Zn) is an essential element in all organisms that plays a fundamental role in numerous cellular functions (Broadley et al., 2007; Cherif et al., 2011). Zn is involved in the catalytic function of many enzymes and structural stability of various cell proteins (Vallee and Falchuk, 1993). Moreover, it has an important role in stabilization and protection of the biological membranes against oxidative stress and the loss of plasma membrane integrity (Aravind and Prasad, 2003). Therefore, Zn deficiency can cause an increase in membrane permeability and a decrease in detoxification mechanisms (Cakmak, 2000). Respect to, it has been shown that the plant roots treated with Cd, an heavy metal that frequently accompanies Zn in the environment, generated oxidative stress, while in combined treatments it was less prominent, indicating that Zn could alleviate oxidative damage (Tkalec et al., 2014). On the other hand, high levels of Zn inhibit many metabolic processes in plants, which can result in limited growth and root development and induce plant senescence (Uruc Parlak and Demirezen Yilmaz, 2012). The extent of Zn phytotoxicity varies in a wide range but mostly depends on plant species, age, environmental conditions, and combinations with other heavy metals (Tsonev and Lidon, 2012). Consequently, the study of the interaction of simple systems with Zn^2+^ may be relevant and a starting point for future studies with other bivalent ions in relation to abiotic stress and the DGPP and PA signaling.

Although, several aspects of DGPP are unknown, it has been shown that the effective lipid molecular shape and the ionization properties of the phosphomonoester head group of DGPP are similar to PA (Kooijman et al., 2007; Kooijman and Burger, 2009). Thus, the ionization properties of the phosphomonoester of DGPP mimic those of PA following the electrostatic-hydrogen bond switch model (Strawn et al., 2012). However, DGPP is not a cone-shaped lipid, i.e., it is not capable of imparting negative curvature stress to the membrane that could facilitate the insertion of hydrophobic protein domains in the membrane (Strawn et al., 2012).

Little is known on the interaction of PA with DGPP and how these are affected by pH and divalent cations, whether these lipids can molecularly mix, or undergo interactions that may modify their individual properties. Within the context briefly reviewed above, it becomes important to understand details of the effects of Zn^2+^ and pH on the surface packing and electrostatic behavior of DGPP, and on its interaction with PA. In a previous study with Langmuir monolayers, we demonstrated that the packing and electrostatic properties of films of pure DGPP and PA were affected by the subphase pH as a consequence of changes in the ionization state of the molecules and that the lipids molecularly mix to form closely packed monolayers at basic pHs (Villasuso et al., 2010). In the present work, we provide further evidences on the molecular packing, in-plane elasticity, and surface electrostatic of films of pure DGPP and 1-palmitoyl-2-oleoyl-*sn*-glycerol 3 phosphate (POPA) and their mixtures on subphases with ZnCl_2_ at different pHs. In addition, we show how the cation affects the monolayer organization by using fluorescence and Brewster angle microscopy (BAM).

## MATERIALS AND METHODS

Langmuir monolayers of the individual lipids and their binary mixtures were spread from premixed solutions in chloroform/methanol (2:1, v/v) onto different subphases at a molecular area larger than the lift off area. Before isometric compression of the film, the solvent was allowed to evaporate for 5 min. All experiments were performed at 24 ± 1°C. Temperature was maintained within ±1°C with a refrigerated Haake F3C thermocirculator and air-conditioning the room temperature. DGPP, POPA, and the lipophilic, fluorescent probe l-α-phosphatidylethanolamine-*N*-(lissaminerhodamine B sulfonyl)-ammonium salt were purchased from Avanti polar lipids Inc. (Alabaster, AL, USA). The lipids were dissolved in chloroform/methanol (2:1, v/v) to a final concentration of 1 nmol μL^-1^. In all the experiments, the subphase was 150 mM NaCl, 5 mM EDTA, pH 8 or 5, with or without 8 mM ZnCl_2_. The pH was stable during the time of the experiment. The subphase was prepared with ultra-pure water obtained from a Millipore purification system (18.2 MΩ). Solvents were of the highest available purity from Merck (Darmstadt Germany).

Surface pressure and surface potential versus molecular area isotherms were obtained at 24 ± 1°C in a Teflon trough of a Langmuir film balance (Monofilmmeter, Mayer Feintechnik, Germany).

Surface pressure was measured with a platinized-PtWilhelmy plate. The surface potential measurements were carried out with a high-impedance millivoltmeter connected to a surface ionizing ^241^Am electrode positioned 5 mm above the monolayer surface and to a reference Ag/AgCl/Cl^-1^ (3 M) electrode connected to the aqueous subphase.

Absence of surface-active impurities in the subphase and in the spreading solvents was routinely controlled as described elsewhere (Maggio, 2004). At least triplicate monolayer isotherms were obtained and averaged at a compression rate of 0.45–0.60 nm^2^ molecule^-1^ min^-1^; for each mixture, it was ascertained that reducing the compression speed produced no change in the isotherms and that recompression after 5 min equilibration of the expanded isotherm at surface pressures below 2 mN m^-1^ led to no significant changes of the limiting mean molecular areas which rules out film loss or kinetically limited artifacts.

Reproducibility was within a maximum SEM of ±1 mN m^-1^ for surface pressure, ±30 mV for surface potential, and ±0.04 nm^2^ for molecular areas. The monolayers of the pure components and of all mixed films were stable and reproducible by recompression.

The monolayer compressibility modulus (κ), also known as in-plane elasticity, was calculated for the pure monolayers and for each mixture in the different conditions as κ = -A_m_(∂π/∂A_m_)_T_. The effect of ZnCl_2_ in the subphase as well as the interactions and molecular miscibility were ascertained from the behavior of the mean molecular area, of the compressibility modulus, and of the average dipole potential per unit of molecular surface density (ΔV⋅*A*, being ΔV the surface (dipole) potential and *A* the mean molecular area at a defined surface pressure); this magnitude is directly proportional to the overall resultant dipole moment in the direction perpendicular to the interface (Gaines, 1966).

The surfaces of the films were observed by fluorescence microscopy (FM), while simultaneously registering the surface pressure vs. molecular area isotherms. The setup consisted of an automated Langmuir balance (KIBRON microtrough) with a Wilhelmy plate for surface pressure determination, mounted on the stage of a Zeiss Axiovert 200 (Carl Zeiss, Oberkochem, Germany) fluorescence microscope with a CCD video camera Zeiss commanded through the Axiovision software of the Zeiss microscope. Long-distance 20× and 40× objectives were employed. Monolayers with different mole fractions of lipids were spread from chloroform/methanol (2:1, v/v) solutions onto different subphases at a molecular area larger than the lift off area. The fluorescent probe was incorporated in the lipid solution before spreading (1 mol %). Before isometric compression of the film, the solvent was allowed to evaporate for 5 min. All experiments were performed at 24 ± 1°C. This method allows analyzing the presence of micron-sized domains of lipids in different phase state and thus, studying bidimensional phase transitions and phase diagrams of mixtures in different conditions (Rosetti et al., 2010; Vega Mercado et al., 2011; Mangiarotti et al., 2014).

For the BAM experiments, an EP3 Imaging ellipsometer (Accurion, Goettingen, Germany) with a 20× objective was used. These observations were performed in order to ensure that the textures on the micron scale observed in FM experiments were not a consequence of the presence of the fluorescent probe; the latter was reported for some systems (Rosetti et al., 2010), while in others the same texture was observed with both techniques (Mangiarotti et al., 2014). All the experiments were performed at 24 ± 1°C.

## RESULTS

In a previous article, the surface behavior of monolayers prepared with DGPP and POPA was described at different pHs. The mixture of both lipids was also analyzed on subphases at basic pHs. As mentioned above, the polar head group of DGPP is a pyrophosphate moiety, and the net charge on the molecule change with the subphase pH (Villasuso et al., 2010). Thus, the polar head group of DGPP may bear from 1 to 3 negative charges, depending on pH (Strawn et al., 2012). Taking the pyrophosphoric acid p*K*a’s in consideration, we performed compression isotherms on subphases with or without ZnCl_2_ at pH 5 (pyrophosphate moiety with 1 or 2 negative charges), and at pH 8 (pyrophosphate moiety with 2 or 3 negative charges) with the aim of analyzing the effect of Zn^2+^on the molecular packing behavior of DGPP and POPA monolayers in different ionization states.

**FIGURE 1:**
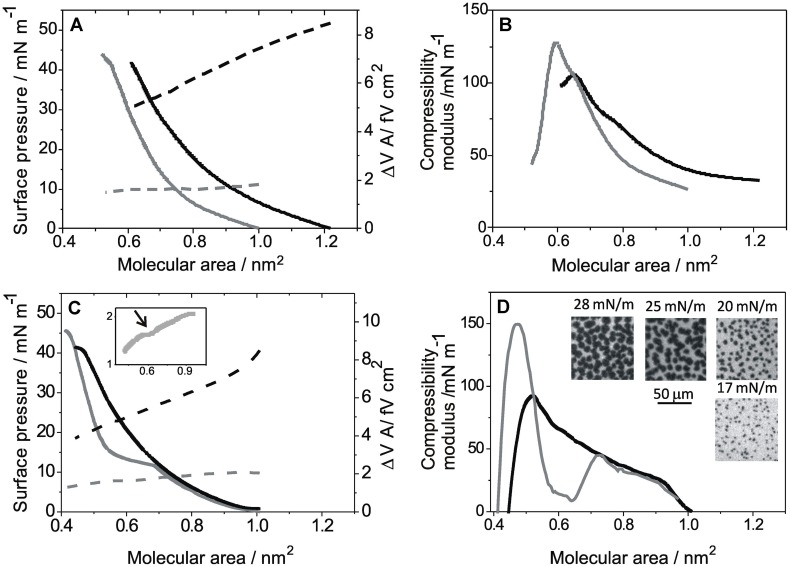
**Compression isotherms for monolayers of diacylglycerol pyrophosphate (DGPP) and palmitoyl-2-oleoyl-*sn*-glycerol 3 phosphate (POPA) on subphases at pH 5: **(A)**** Lateral pressure (solid lines, left scale) and ΔV⋅A (dashed line, right scale) as a function of the mean molecular area for DGPP monolayers. **(B)** Compressibility modulus for the isotherms shown in **A**. **(C)** Lateral pressure (solid lines, left scale) and ΔV⋅A (dashed line, right scale) as a function of the mean molecular area for POPA monolayers. Inset: zoom for ΔV⋅A of POPA on solutions with Zn^2+^ (same as in the main plot). **(D)** Compressibility modulus for the isotherms shown in **C**. Insets: representative images for POPA films on solutions with Zn^2+^ obtained with FM at the indicated lateral pressures. Real size: 100 μm × 100 μm. Subphase composition: 0.15 M NaCl + 5 mM EDTA, pH 5 (black lines) and 0.15 M NaCl, 5 mM EDTA + 8 mM ZnCl_2_, pH 5 (gray line). All curves show a single representative experiment from a set of triplicates.

**Figure 1** shows the compression isotherms of DGPP (A) and POPA (C) on subphases at pH 5 with and without 8 mM ZnCl_2_. The compression isotherms in the absence of ZnCl_2_ have been previously reported (Villasuso et al., 2010). Briefly, both lipids formed expanded monolayers with compressibility moduli increasing continuously up to about 100 mN m^-1^ at collapse (**Figures 1B,D**, black lines), which occurred at about 40 mN m^-1^ for both lipids on acid subphases.

The presence of Zn^2+^ in the subphase caused a shift of the whole isotherm of DGPP to smaller average molecular area (**Figure 1A**, solid gray line) with a concomitant increase of the compressibility modulus at high molecular densities (**Figure 1B**, gray line), without an appreciable change of the collapse pressure (collapse point: 42 mN m^-1^ at 0.58 nm^2^). The lift off area with zinc in the subphase was 1.05 nm^2^. The compressibility modulus ranged from 25–30 mN m^-1^ to 130 mN m^-1^, indicating that in the presence of Zn^2+^, DGPP also formed monolayers with a liquid-expanded behavior (Davies and Rideal, 1963) but with a more condensed character under compression compared to the behavior on subphases without Zn^2+^.

In order to explore the electrostatics of these monolayers, the surface potential was determined in the absence and in the presence of Zn^2+^. The cation induced a three to fivefold diminution in ΔV⋅*A* depending on the film packing (**Figure 1A**, dotted lines).

For POPA, the presence of zinc in the subphase induced a phase transition from a liquid-expanded to a liquid-condensed state at about 11.5 mN m^-1^ (**Figure 1C**, solid gray line). The phase transition was clearly observed as a minimum in the compressibility modulus (**Figure 1D**, gray line). The condensed state showed compressibility moduli up to 150 mN m^-1^ at collapse (**Figure 1D**, gray line), corresponding to a liquid-condensed character (Davies and Rideal, 1963). The phase transition induced by Zn^2+^ probably reflects a decrease of lateral repulsion in the charged polar head groups caused by the divalent cation as previously observed for POPA monolayers in the presence of Mg^2+^(Brockman et al., 2003).

The Zn^2+^ induced phase transition corresponded to a first-order, phase transition since micron-sized domains formed by lipids in the condensed state were observed in the two-phase coexistence region using BAM and FM. In **Figure 1D**, representative images for POPA on solutions with Zn^2+^ at the indicated surface pressure obtained with FM are shown; similar images were obtained using BAM while in the absence of Zn^2+^ domains were not observed at any surface pressure (data not shown).

**FIGURE 2:**
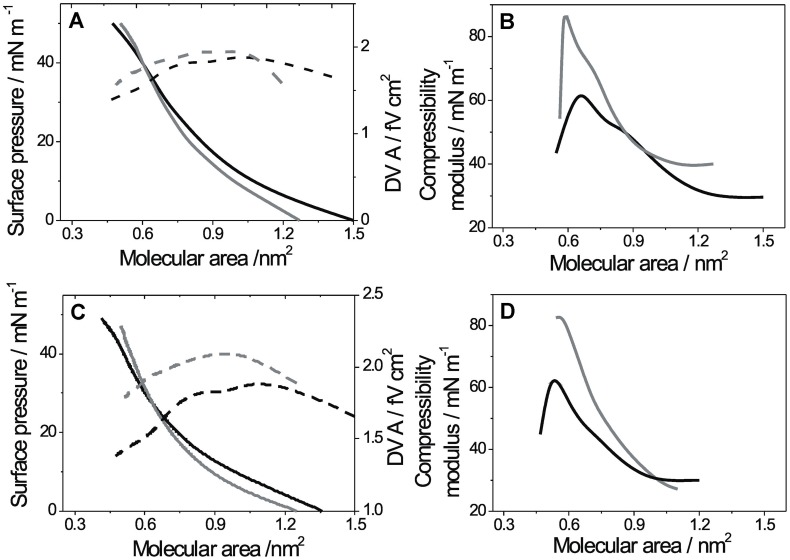
**Compression isotherms for monolayers of DGPP and POPA on subphases at pH 8: **(A)**** Lateral pressure (solid lines, left scale) and ΔV⋅A (dashed line, right scale) as a function of the mean molecular area for DGPP monolayers. **(B)** Compressibility modulus for the isotherms shown in **A**. **(C)** Lateral pressure (solid lines, left scale) and ΔV⋅A (dashed line, right scale) as a function of the mean molecular area for POPA monolayers. **(D)** Compressibility modulus for the isotherms shown in **C**. Subphase composition: 0.15 M NaCl + 5 mM EDTA, pH 8 (black lines) and 0.15 M NaCl, 5 mM EDTA + 8 mM ZnCl_2_, pH 8 (gray line). All curves show a single representative experiment from a set of triplicates.

The effect of ZnCl_2_ on ΔV⋅A at pH 5 was similar to that observed for DGPP, i.e., Zn^2+^ inducing a 2–4 times decrease depending on the molecular packing density. The phase transition was detected as a change of slope of the ΔV⋅*A* vs. *A* isotherm at about 0.65 nm^2^ (see arrow in **Figure 1C** inset). In **Figure 2**, the effect of Zn^2+^ ions is shown at basic pH. At pH 8, the films of DGPP were loosely packed (Villasuso et al., 2010) and the effect of Zn^2+^was less marked than at acid pH (**Figure 2A**). However, a small condensation of the film could still be observed when the cation was added to the subphase and the compressibility modulus increased from 60 to 80 mN m^-1^ at collapse (**Figure 2B**). A similar effect was induced by Zn^2+^ in POPA monolayers (see **Figures 2C,D**). Regarding the interfacial electrostatics, the presence of Zn^2+^ at this pH induced an increase of ΔV⋅*A*, contrary to the effect observed on subphases at acid pH. The increase of ΔV⋅*A* was of about 20%.

It was shown that the effective molecular shape of PA is highly influenced by pH, temperature, and the presence of divalent cations (Minones et al., 2002; Kooijman et al., 2003, 2005; Villasuso et al., 2010). At pH 7 in the presence of magnesium ion, PA acquires a cylindrical shape and thus stabilizes the bilayer. However, once subjected to an acid environment, the head group of PA decreases its effective size, and the lipid undergoes a shape change to a cone-like shape, thus affecting the membrane by promoting an increase of negative membrane curvature (Kooijman et al., 2003). Diacylglycerol pyrophosphate formation after stimulus takes place after a transient increase of the PA levels (Munnik et al., 1995). As a consequence, a temporary and local accumulation of DGPP and its precursor at the membrane interface should be expected. Since the monolayer packing properties are affected by the interactions of POPA and DGPP at pH 8 (Villasuso et al., 2010), the phospholipid packing may be affected during the signaling processes. Therefore, we studied the packing properties of mixed films of DGPP and POPA in the presence of Zn^2+^ on subphases at acidic and basic pHs.

**FIGURE 3:**
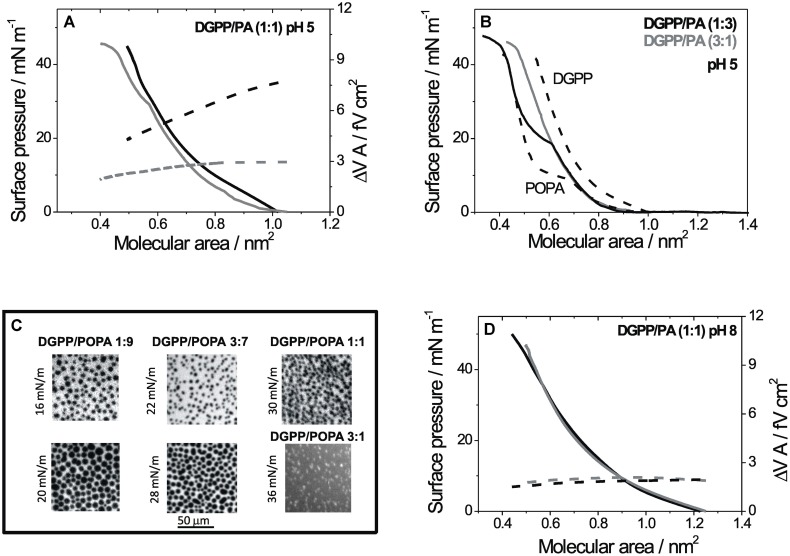
**Compression isotherms for mixed monolayers of DGPP and POPA on subphases at different pHs:** (A)**** Lateral pressure (solid lines, left scale) and ΔV⋅A (dashed line, right scale) as a function of the mean molecular area for monolayers composed of DGPP/POPA (1:1) on subphases at pH 5. **(B)** Lateral pressure as a function of the mean molecular area for monolayers composed pure DGPP and POPA (dashed lines) or mixtures of DGPP/POPA in a proportion (1:3) (black line) and (3:1) (gray line). **(C)** Representative images of mixed films at the indicated proportions and pressures obtained with FM on subphases at pH 5 with Zn^2+^. Real size: 100 μm × 100 μm. **(D)** Lateral pressure (solid lines, left scale) and ΔV⋅A (dashed line, right scale) as a function of the mean molecular area for monolayers composed of DGPP/POPA (1:1) on subphases at pH 8. Subphase composition: 0.15 M NaCl + 5 mM EDTA, pH 8 (black lines) and 0.15 M NaCl, 5 mM EDTA + 8 mM ZnCl_2_, pH 8 (gray line).

**Figure 3** shows the behavior of mixed monolayers of DGPP and POPA when ZnCl_2_ is added to the subphase at pHs 5 and 8. **Figure 3A** shows the compression isotherm for a 1:1 mixture at pH 5 with Zn^2+^ compared with the isotherm in the absence of Zn^2+^. Similar to the pure lipids, the cation decreased the potential from 8–6 to 3–2 fV cm^2^ and a phase transition from a liquid-expanded to a liquid-condensed film was induced. However, the transition occurred at 30 mN m^-1^ and was less marked. As shown in **Figure 2B**, the phase transition of POPA induced by Zn^2+^ was observed at increasing pressures and in a less marked fashion as the proportion of DGPP in the mixtures increased. The fact that the lateral pressure for the phase transition was higher as the proportion of DGPP increased in the film indicates that DGPP mixed preferentially with POPA in the liquid-expanded state, thus stabilizing the latter with respect to the condensed phase. **Figure 3C** shows representative FM images of mixed monolayers at the surface pressures in which the phase coexistence is observed in each case, similar images were obtained using BAM (data not shown). At 75% of DGPP, no domains were observed up to 36 mN m^-1^. Nevertheless, as the monolayer approached the collapse point, the fluorescent probe was segregated in bright spots indicating that the monolayer acquired a more condensed character, expelling the bulky probe (see image in **Figure 3C**).

At pH 8, the surface pressure- and ΔV⋅*A*-mean molecular area compression isotherms for mixed monolayers of DGPP with POPA were only slightly affected by the presence of the divalent cation (see **Figure 3D**).

## DISCUSSION

**FIGURE 4:**
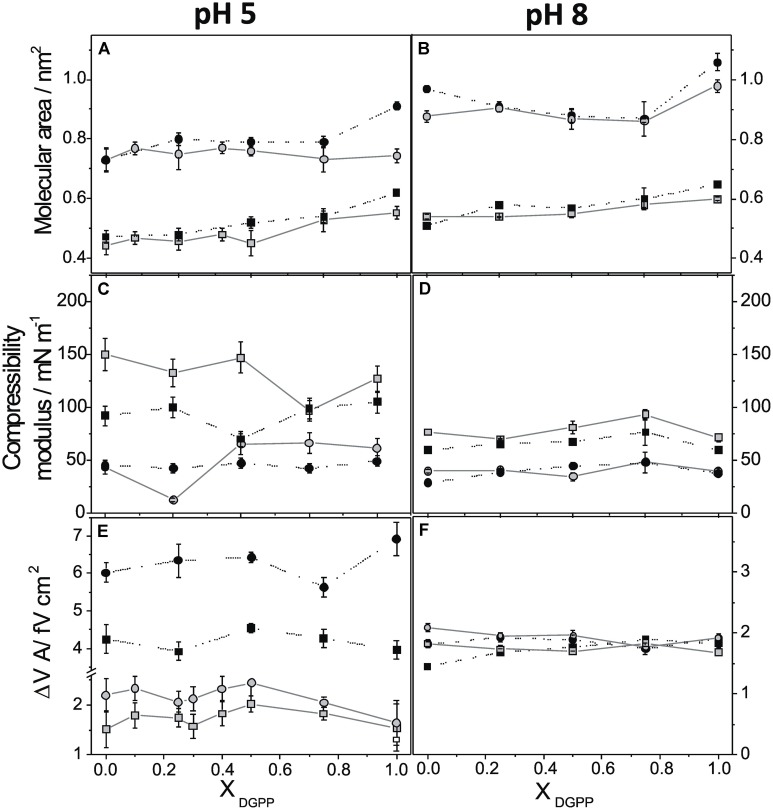
**Mean molecular area (A,B), compressibility modulus (C,D), and surface potential density (E,F) as a function of the mole fraction of DGPP in the mixture at pH 5 (left panel) and pH 8 (right panel).** The lateral pressures are: 10 mN m^-1^ (circles) and 40 mN m^-1^ (squares). Subphase composition: 0.15 M NaCl + 5 mM EDTA, (black circle) and 0.15 M NaCl, 5 mM EDTA + 8 mM ZnCl_2_, (gray circle).

The behavior upon compression of films composed of DGPP, POPA, and their mixtures in the presence of ZnCl_2_ was investigated on acid and basic subphases. **Figure 4** summarizes the effect of the presence of ZnCl_2_ at 10 and 40 mN m^-1^ at pH 5 (lefts panels) compared to pH 8 (right panels). The mean molecular area, the compressibility of the film, and Δ*V*.*A* are shown as a function of the mole fraction of DGPP in mixed films with POPA. The same scale was used for both pHs in panels A–D in order to facilitate comparison. For panels E and F, different scales were used for better understanding of the data.

At pH 5, the molecular density of pure DGPP and of films enriched in this lipid was markedly increased by the divalent cation while the effect was reduced in films with a high proportion of POPA (**Figure 4A**). This effect was more marked at low than at high surface pressure, where the mean molecular area decreased 0.1–0.2 nm^2^. This is probably a consequence that the mean molecular area at 30 mN m^-1^ or higher pressures was close to the minimal area occupied by two hydrocarbon chains (0.38–0.40 nm^2^), and thus the cation was not able to further reduce the area occupied by the lipid. However, slope of the isotherms in the whole range of surface pressures was higher in the presence of Zn^2+^ with a concomitant increase in the surface compressibility modulus. At pH 5, monolayers in the presence of Zn^2+^ were less compressible than in its absence (see **Figure 4C**). Exceptions were observed for the proportions in which the induced phase transition coincides with the lateral pressure analyzed (25% at 10 mN m^-1^ and 75% at 40 mN m^-1^). The compression isotherms of monolayers for a particular lipid species depends on the length and unsaturation of the hydrocarbon chain and on the bulkiness and charge of the polar head group. Long and saturated hydrocarbon chains generally promote more condensed monolayer. In contrast, ionization of the lipid head groups should result in repulsive interaction, leading to loosely packed monolayers (Brown and Brockman, 2007). Thus, the observed compression isotherms of DGPP and POPA at pH 5 in the presence of the cation are in agreement with a decreased electrostatic repulsion where a lower net charge is expected. In similar experiments, it was observed that DPPA monolayers are also influenced by pH as a consequence of the change in the ionization of the lipid (Minones et al., 2002). At pH 3, DPPA molecules are uncharged showing a more rigid monolayer structure compared to monolayer spread on subphases at higher pHs. It is possible that hydrogen bonding between protonated PA groups of adjacent molecules could lead to a more stable and coherent monolayer. In contrast, DPPA monolayer at pH 6 exhibits a larger molecular area due to the ionization of the first phosphate of PA, decreasing the possibility for intermolecular hydrogen bonding. The effect of divalent cations on the interfacial behavior of anionic lipid was also evaluated with other phospholipids in model system. Apparently some divalent cations at high concentration may induce the formation of clusters of phospholipids. In the case of the precursor of second messenger PIP_2_, only Ca^2+^, but not Mg^2+^, Zn^2+^, or polyamines induces a surface pressure drop that coincides with the formation of PIP_2_ clusters (Wang et al., 2012). Our results with Zn^2+^ at high concentration (ca. 3 mM free ion) indicated that the ion promotes the generation of domains of POPA in a condensed phase state. Which is the lower amount of this ion capable of induce a phase transition in monolayers of this lipid is however, not investigated, since it is beyond the scope of this research.

At pH 8, only the monolayers composed of pure DGPP or POPA were condensed by Zn^2+^, while the film density of the mixed monolayers was unaffected by this cation (**Figure 4B**). This is probably a consequence of the fact that the mixture at basic pHs forms closely packed films and the process of mixing is thermodynamically favored (Villasuso et al., 2010), therefore, the Zn^2+^ ions are probably not able to penetrate and further modify the compact film formed by the mixture. The compressibility modulus as a function of the proportion of DGPP in the mixture at pH 8 is shown in **Figure 4D**, and the comparison with **Figure 4C** indicates that at this pH only a slight increase of the compressibility was observed at 10 mN m^-1^ while the effect was negligible at 40 mN m^-1^.

Regarding the surface electrostatics, **Figures 4E,F** show that the ΔV⋅*A* of the pure lipids and their mixtures changed from 4–6 to 2 fV cm^2^ when the pH increased. The negative charge on the lipids signifies an anionic surface with a corresponding distribution of mobile ions in the immediate aqueous milieu (the double-layer potential) which is also included in the surface potential measured. It was previously observed in stearic acid monolayers that an increase of the negative charge in the monolayer may decrease the double-layer potential by values as high as 200 mV (Vega Mercado et al., 2011). The surface potential of charged films depends in a rather complex manner on the double-layer potential because of the simultaneous contribution of hydrocarbon chain dipoles, of water dipoles from the polar head group hydration shell, and intrinsic polar head group dipoles, as well as from the electrostatic field brought about from the relative position of mobile ions close to the monolayer. Therefore, a decrease of the double-layer potential when the lipid becomes ionized may result in a lower surface potential even if the overall lipid dipole is being increased in the negatively charged molecule (Vega Mercado et al., 2011). Thus, the decrease of ΔV⋅*A* observed at pH 8 compared to pH 5 could most probably be a consequence of the decrease of the double-layer potential as the monolayer charge increased. Studies of the interaction between PA and Ba^2+^ suggested that PA is extremely effective in binding divalent ions through its oxygen atoms, with a broad distribution of binding constants and exhibiting the phenomenon of charge inversion (a total number of bound counterion charges that exceeds the negative PA charge; Faraudo and Travesset, 2007). The authors predict that a PA-rich domain undergoes a drastic reorganization when divalent cations as Ca^2+^ and Ba^2+^ reach micromolar concentrations (i.e., typical physiological conditions), as PA lipids become doubly charged by releasing their protons. Although restricted to PA, those results could be qualitatively similar for other phospholipids, as DGPP, which play somewhat similar roles as PA.

When Zn^2+^ was present in the subphase, the measured ΔV⋅*A* was about 2 fV cm^2^ at both pHs. This leads to the conclusion that this parameter is very sensitive to the Zn^2+^ cation at pH 5 and at all pressures, while at pH 8 only the pure POPA monolayers were affected by the cation and, in a less marked manner than at pH 5. Besides, at pH 8 the interface was slightly depolarized while at pH 5 it became highly hyperpolarized.

The relatively low response of the surface electrostatics at pH 8 may be explained by considering that in this condition, the charge density of the interface was quite negative, and a compact, double layer was already formed in the absence of Zn^2+^. The addition of this cation may not induce further changes in the system, in spite of any specific binding with the film molecules. Also, it is possible that at basic pHs, Zn^2+^ did not interact specifically with the film and thus, only changed slightly the ionic strength of the subphase with correspondingly small changes in the film properties (**Figures 4B,D,F**).

At pH 5, on the other hand, the divalent cation appeared to interact with the film and to induce changes in the film properties studied (**Figures 4A,C,E**). The important changes induced by Zn^2+^ clearly indicate an interaction with the phosphate groups, decreasing the in-plane repulsion and hyperpolarizing the interface. How this interaction may translate to changes in ΔV⋅*A* is not easy to explain because this parameter depends on the location of Zn^2+^ ions relative to the polar head groups and to the interface.

## CONCLUSION

Our results indicate that the film properties of DGPP and its precursor PA were sensitive to Zn^2+^ in a pH-dependent manner, and thus depend on the ionization state of the molecules forming the film. This effect was further modulated by the relative proportions of DGPP and POPA in the film. The changes promoted by Zn^2+^ were more marked on subphases at pH 5, while at pH 8 the influence of the cation in the film surface properties was attenuated.

Regarding the ionization behavior of PA, it was recently proposed that upon initial ionization, the remaining hydrogen may form hydrogen bonds with this phosphomonoester head group, resulting in an easier deprotonation compared to the situation lacking the hydrogen bond. This peculiar ionization behavior was summarized in the electrostatic-hydrogen bond switch model. Similarly, the phosphomonoester head group of DGPP was proposed to follow this behavior. In addition, it was observed that at constant pH, DGPP carries more negative charge than the phosphomonoester head group (Strawn et al., 2012). Strawn et al. (2012) suggested that the higher charges would favor the interaction of positive domains of proteins and might result in a displacement of a PA-bound protein to DGPP. If in the complex mixture, POPA and DGPP also form a stable film when locally mixed, then in the presence of DGPP, POPA would not be as free as in its absence. Furthermore, the interaction of both molecules with Zn^2+^ ions would be precluded in the mixed film.

If the surface behavior described could happen in biomembranes, it may be speculated that such effects could occur locally when DGPP is generated by PAK or hydrolyzed by DGPP phosphatase. In this work, we demonstrate that a local increase in the proportion of this lipid can affect the local film properties, in a pH-dependent manner which is also sensitive to the ionic composition of the aqueous milieu. Taking together, all these effects may constitute structural-electrostatic signal transduction mechanism involving DGPP in the turning off/on of PA signaling.

## AUTHOR CONTRIBUTIONS

Ana L. Villasuso and Natalia Wilke conceived and designed the experiments. Ana L. Villasuso performed the experiments. Ana L. Villasuso and Natalia Wilke analyzed the data. Estela Machado and Bruno Maggio contributed reagents/materials/analysis tools. Ana L. Villasuso, Natalia Wilke, Estela Machado, and Bruno Maggio involved in the Result discussion. Ana L. Villasuso, Natalia Wilke, Estela Machado, and Bruno Maggio wrote the paper. Ana L. Villasuso, Natalia Wilke, Estela Machado, and Bruno Maggio edited the manuscript, wrote figure legends, and helped with the outline of the paper:.

## Conflict of Interest Statement

The authors declare that the research was conducted in the absence of any commercial or financial relationships that could be construed as a potential conflict of interest.
